# Doping Detection Based on the Nanoscale: Biosensing Mechanisms and Applications of Two-Dimensional Materials

**DOI:** 10.3390/bios15040227

**Published:** 2025-04-03

**Authors:** Jingjing Zhao, Yu Wang, Bing Liu

**Affiliations:** Shanghai Institute of Doping Analyses, Shanghai University of Sport, Shanghai 200438, China; zhaojingjing@sus.edu.cn (J.Z.); wyy202423@163.com (Y.W.)

**Keywords:** doping detection, two-dimensional materials, nanoscale, MOFs, biosensing mechanisms

## Abstract

Doping undermines fairness in sports and threatens athlete health, while conventional detection methods like LC-MS and GC-MS face challenges such as complex procedures, matrix interferences, and lengthy processing times, limiting on-site applications. Two-dimensional (2D) materials, including graphene, MoS_2_, and metal–organic frameworks (MOFs), offer promising solutions due to their large surface areas, tunable electronic structures, and special interactions with doping agents, such as hydrogen bonding, π-π stacking, and electrostatic forces. These materials enable signal transduction through changes in conductivity or fluorescence quenching. This review highlights the use of 2D materials in doping detection. For example, reduced graphene oxide–MOF composites show high sensitivity for detecting anabolic steroids like testosterone, while NiO/NGO nanocomposites exhibit strong selectivity for stimulants like ephedrine. However, challenges such as environmental instability and high production costs hinder their widespread application. Future efforts should focus on improving material stability through chemical modifications, reducing production costs, and integrating these materials into advanced systems like machine learning. Such advancements could revolutionize doping detection, ensuring fairness in sports and protecting athlete health.

## 1. Introduction

In the context of the highly competitive sports environment, the use of performance-enhancing substances has become a significant issue that threatens both the fairness of sports and the health of athletes [1]. The use of doping not only undermines the integrity of competitions but may also have long-term negative impacts on athletes’ physical health [2]. Although the World Anti-Doping Agency (WADA) has implemented various policies and measures to regulate athletes, reports of doping remain prevalent, with many athletes resorting to such substances in pursuit of improved performance or enhanced stimulation [3,4,5]. Therefore, the effective detection and control of doping substances has become an urgent task that requires the concerted efforts of both the sports and scientific communities.

Traditional methods for detecting doping, such as liquid chromatography–mass spectrometry (LC-MS) [6] and gas chromatography–mass spectrometry (GC-MS) [7], while offering high sensitivity, require complex sample preparation [8] and are easily interfered with by the sample matrix. Furthermore, these methods typically involve lengthy processing times, making on-site, real-time testing during competitions or athletes’ training sessions difficult [9,10]. This increases the risk of unethical individuals attempting to cheat during the testing period. Moreover, with new doping strategies constantly emerging, traditional detection methods are struggling to keep up and failing to meet the ever-increasing demands of testing. Accurate and timely detection of doping is essential, as it not only helps identify violations quickly but also serves as a strong deterrent against potential doping violations, reinforcing the fairness, integrity, and purity of sports competitions. Therefore, the development and application of nanoscale detection technology offer great potential for advancing regulatory management and strengthening the integrity of the sports sector.

**Figure 1:**
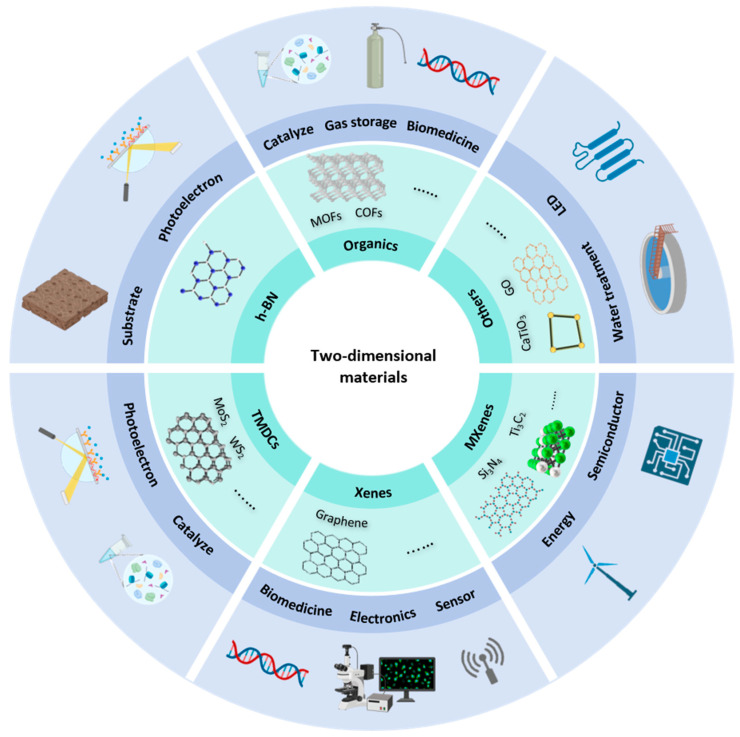
Categories, properties, and applications of two-dimensional materials.

Two-dimensional (2D) materials are a class of layered materials composed of one or more layers of inorganic or organic molecules or polymers [11], usually with a thickness of a few nanometers in the vertical direction [12]. The interaction between layers is mainly driven by van der Waals forces, so the layers can be effectively peeled and stacked during the manufacturing process, and the thickness is expressed in angstroms when materials reach the atomic level [13]. As shown in Figure 1, 2D materials can be divided into six categories: single-element 2D materials (Xenes), transition metal sulfides (TMDCs), hexagonal boron nitride (h-BN), MXenes, 2D organic materials, and other 2D materials [14]. Typical single-element materials include graphene, black sintering, silicene, etc., which are composed of a single element to form a layered structure. The chemical formula of Xenes is MX2, where M is a transition metal, commonly Mo and W, and X is a chalcogen element, such as S, Se, and Te. Two-dimensional organic materials are further divided into metal–organic frameworks (MOFs) and covalent organic frameworks (COFs), the first coordinated by metal ions and the second connected by covalent bonds. The development of two-dimensional materials is changing with each passing day. Common ones include two-dimensional oxides, two-dimensional perovskites, and two-dimensional heterojunctions. Their physical and chemical properties in two-dimensional structures are different from those in three-dimensional structures. Researchers have explored their excellent performance in the field of optoelectronics, and they have been widely used in devices such as thin-film transistors, solar cells, organic light-emitting diodes (OLEDs), and sensors [15,16,17,18,19,20,21,22,23]. Two-dimensional materials have great application potential in flexible electronic devices, photoelectric conversion, and information storage [24,25,26,27]. This article focuses on the application of two-dimensional materials and detection, especially doping detection, and attempts to improve detection efficiency using two-dimensional materials to enrich small molecules or macromolecules and to improve the signals of common instruments. The development of sensors in biomedicine is a fast-growing area, where two-dimensional materials are used to design wearable devices or sensors, converting the test object into photoelectric signals, improving the response speed, and focusing on real-time detection [28]. In the field of doping detection, most of the electrochemistry and two-dimensional materials are combined to improve sensitivity [28,29,30,31].

This review focuses on the detection of doping at the nanoscale, with a particular emphasis on the biosensing mechanisms and applications of 2D materials. It explores their binding mechanisms and selective recognition processes, as well as the intrinsic interactions between 2D materials and doping molecules. Additionally, the review discusses the development prospects of these materials for doping detection.

## 2. Characteristics of 2D Materials

### 2.1. Structure and Properties

Detection of doping at the nanoscale is gradually becoming an important research direction in the field of biomedical science [32,33]. In particular, 2D materials exhibit great potential due to their unique physical and chemical properties. With atomic-level thickness and a 2D planar structure, they offer exceptional surface sensitivity and selectivity, making them ideal platforms for biosensing applications [34,35,36,37,38].

**Figure 2:**
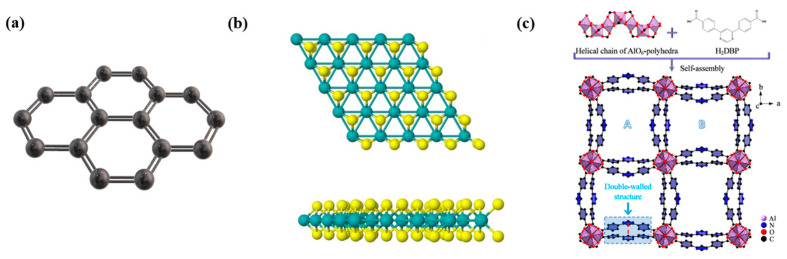
Schematic diagram of (**a**) graphene, (**b**) MoS_2_ [45], and (**c**) double-walled Al-based MOFs [46]. Reprinted with permission from references [45,46].

Graphene is a prominent representative of 2D materials, renowned for its unique structure and remarkable properties. As shown in Figure 2a, it consists of carbon atoms arranged in a hexagonal honeycomb lattice, with each carbon atom bonded to three neighboring carbon atoms through sp^2^ hybridized orbitals, forming σ-bonds with equal bond lengths of approximately 0.142 nm and bond angles of 120°. This highly symmetric arrangement results in a regular hexagonal lattice that extends across the entire plane [39,40,41]. The strong covalent bonds between the carbon atoms impart significant structural stability to the material [42]. Additionally, each carbon atom retains one unhybridized p-electron, which is orthogonal to the graphene plane. These p-electrons form off-plane π-bonds, which are delocalized over the entire structure [43]. The presence of these off-plane π-bonds not only plays a crucial role in the electrical and optical properties of graphene but also facilitates interactions with other molecules via π-π stacking interactions [44].

MoS_2_, a transition metal sulphone, has a unique crystal structure. As shown in Figure 2b, it consists of three atomic layers arranged in a “sandwich” configuration, with molybdenum (Mo) atoms residing in the central layer, while sulfur (S) atoms form the upper and lower layers [47,48]. These atoms are firmly bonded by covalent interactions within each layer, forming a stable and orderly structure [45]. The interlayer interactions, however, are governed by weaker van der Waals forces, allowing for the easy exfoliation of MoS_2_ into monolayers or few-layer structures. This layered structure endows MoS_2_ with remarkable surface characteristics, where the exposed Mo and S atoms act as active sites. These sites are highly reactive and can engage in specific interactions with biomolecules, functioning like “keys” that selectively bind to corresponding “locks” on biomolecules [49,50]. Such interactions occur through ligand binding or electrostatic forces, facilitating the attachment of proteins, nucleic acids, and other biomolecules to the MoS_2_ surface. This selective binding capability enhances the sensitivity and specificity of MoS_2_-based biosensors, providing a reliable foundation for biosensing applications [51,52]. In addition to its surface reactivity, MoS_2_ also exhibits excellent chemical stability. Under appropriate conditions, MoS_2_ maintains its structural integrity and performance across a variety of complex biological environments, which is essential for the sustained function of biosensors [53]. This stability ensures that MoS_2_-based sensors can operate reliably over extended periods, thereby expanding their applicability in practical detection scenarios, including doping detection, where accuracy and long-term stability are paramount. Consequently, MoS_2_ holds significant promise as a material for advancing biosensor technologies, offering both high performance and versatility in real-world applications [54,55].

Metal–organic frameworks (MOFs) represent a class of advanced materials in the field of materials science distinguished by their porous, periodic network structures formed through the self-assembly of metal ions or metal clusters with organic ligands [46]. The intricate structural characteristics of MOFs confer exceptional physicochemical properties, which make them highly suitable for a wide range of applications, particularly in the realm of biosensing. One of the most notable features of MOFs is their extremely high porosity and specific surface area. The internal pore structure of MOFs is both complex and highly developed, with specific surface areas that can reach several thousand square meters per gram [56]. This high surface area renders MOFs highly effective as “molecular adsorption platforms”, enabling them to adsorb a large variety of molecules, including biomolecules. The double-walled aluminum-based MOF shown in Figure 2c has a micropore specific surface area of 2235 m^2^/g, and its excellent chemical stability enables it to effectively adsorb trace amounts of benzene [46]. The abundance of available binding sites on the MOF surface significantly enhances the sensitivity of biosensors, allowing for the precise capture and detection of even trace amounts of biomolecules. This feature is particularly advantageous in applications where high detection sensitivity is required, such as in the detection of low-abundance analytes [57,58]. In addition to their high porosity, MOFs offer remarkable design flexibility [59]. The structure of MOFs can be tailored at the molecular level by selecting different metal ions and organic ligands, which confers a high degree of customization in terms of both structure and function. This structural tunability allows for the design of MOFs with specific properties suited to particular applications [60]. For instance, the incorporation of organic ligands with biomolecular recognition capabilities enables MOFs to act as “molecular-specific probes”, capable of selectively binding to target biomolecules. This selectivity improves the specificity of biosensors, effectively minimizing interference from non-relevant molecules and ensuring the accuracy and reliability of biosensing assays [61,62,63]. Furthermore, MOFs exhibit a range of physicochemical properties that can be leveraged for biosensing applications. Depending on the choice of metal ions and organic ligands, MOFs can display fluorescence, magnetism, and other properties that enhance their functionality [64,65]. These characteristics provide multiple detection modalities, such as fluorescence-based real-time monitoring of biomolecular dynamics and the use of magnetic properties for the rapid separation and enrichment of biomolecules. These capabilities not only improve the sensitivity and efficiency of biosensing but also facilitate the development of more sophisticated detection strategies for real-time, high-throughput applications [66,67]. Therefore, the combination of high porosity, structural design flexibility, and multifunctional physicochemical properties makes MOFs an ideal material for advancing biosensor technologies.

This review primarily focuses on the application of two-dimensional materials in the field of stimulant detection. The properties of other two-dimensional materials that have not been applied in stimulant detection will not be discussed further in this review.

### 2.2. Advantages of Applications in Biosensing

As biosensor technologies continue to evolve, 2D materials are poised to play a central role in the next generation of diagnostic devices, providing innovative solutions for a wide array of applications in medicine, environmental monitoring, and beyond. The combination of advanced materials and nanotechnology is expected to drive significant advancements in the speed, accuracy, and scalability of biosensing platforms, positioning them as essential tools for addressing global health challenges.

**Figure 3:**
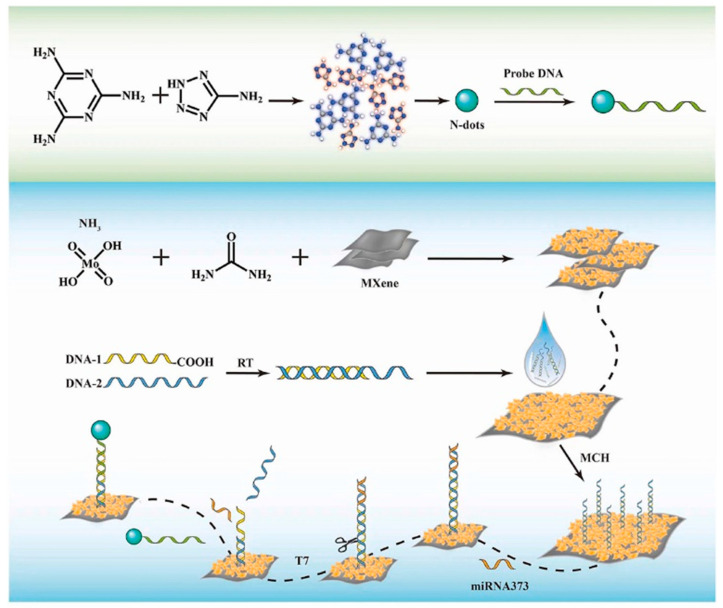
Van der Waals heterojunction-based biosensors can effectively enhance the ECL signal at the N-point to detect miRNA-373 in the range of 1 fM~1 μM, improving sensitivity. Reprinted with permission from reference [73].

From the perspective of high sensitivity, the unique atomic-scale thickness and 2D planar structure of 2D materials confer exceptional properties, including an ultra-high specific surface area and a wealth of active sites [25,35]. These characteristics significantly enhance their ability to interact with and detect biomolecules, making them ideal candidates for sensitive biosensing applications. For instance, graphene, with its theoretical specific surface area exceeding 2600 m^2^/g [68], provides an expansive surface for the adsorption of biomolecules. This extensive adsorption capacity allows for the enrichment of biomolecules at the surface, thereby amplifying the detection signal and enhancing the sensitivity of biosensors [69,70,71]. Similarly, the active sites on the surface of MoS_2_ offer highly specific interactions with biomolecules, functioning as precise molecular “grasping hands” that can selectively capture target molecules [72]. These interactions are particularly advantageous for detecting low-concentration biomarkers or drug components, even in trace amounts. Molybdenum disulfide-based sensors exhibit high sensitivity, with a detection range capable of reaching femtomolar levels (as shown in Figure 3), accurately detecting trace amounts of biomolecules, which is crucial for applications that require the detection of trace substances in biological samples or drug testing [73]. Such capabilities are particularly relevant for early disease diagnosis and pharmacokinetic studies, where the ability to detect small amounts of biomarkers or pharmaceutical compounds can provide valuable insights into disease progression and drug development [72,73,74,75]. These materials, such as graphene [76] and molybdenum disulfide (MoS_2_) [77], with their ultra-high surface area and specific molecular interactions, are thus poised to play a pivotal role in advancing the field of biosensing, providing robust technical support for early disease diagnosis, personalized medicine, and the monitoring of drug efficacy in the context of pharmaceutical research and development. By refining sensor architecture and experimental protocols, detection limits for these materials have reached the picomolar and even femtomolar levels, surpassing the performance of traditional detection methodologies [78,79,80]. For instance, a research team developed an electrochemical sensing platform integrating molybdenum disulfide with carbon nanodots, achieving ultra-sensitive detection of salicylic acid, catechol, and resorcinol at trace levels [81]. This platform demonstrated exceptional sensitivity and the ability to detect multiple substances simultaneously, underscoring the value of 2D materials in complex analytical contexts.

**Figure 4:**
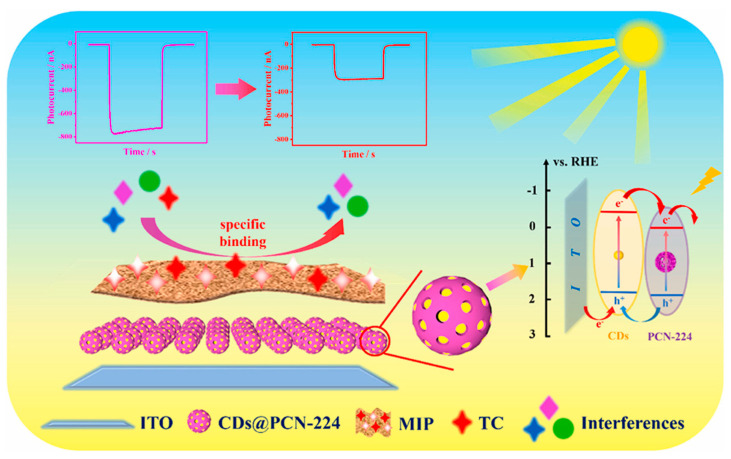
A novel MOF pore domain-confined quantum dot photosensitive material was designed and synthesized to construct a novel MIP-PEC cathode sensor for fast and sensitive TC detection. Reprinted with permission from reference [84].

In terms of high specificity, highly selective recognition of target biomolecules or drug molecules can be achieved through the strategic design of the structure and ligands of MOFs, as well as through meticulous surface modification of materials such as zinc oxide (ZnO) [82,83]. By incorporating ligands with tailored recognition capabilities, MOFs can be engineered to selectively capture target molecules, such as proteins, nucleic acids, or metabolites, functioning as customized “molecular locks” that facilitate highly specific molecular interactions. The cavities in the molecularly imprinted polymers shown in Figure 4 can specifically recognize the target, blocking electron transfer and “turning off” the response signal, thereby achieving selective detection of tetracycline (TC). This design approach significantly reduces the likelihood of non-specific binding, thereby minimizing interference from structurally similar molecules and enhancing the precision of the detection process [84]. This highly selective binding is particularly valuable when detecting specific targets in complex biological matrices, where the presence of numerous other biomolecules may otherwise complicate detection efforts. The high specificity of these materials ensures the accuracy and reliability of biosensing assays, which is critical in reducing false-positive and false-negative results—issues that can have significant consequences in clinical diagnostics, forensic analysis, and personalized medicine [85,86,87]. In applications such as clinical diagnostics, where accurate identification of biomarkers is essential for disease detection and monitoring, or in forensic identification, where the precise analysis of biological samples is required, the ability to achieve highly specific recognition of target molecules is crucial. Moreover, in the field of personalized medicine, where treatment strategies are tailored to the individual patient’s molecular profile, the use of highly specific biosensors can enable more accurate drug monitoring and therapeutic interventions. Therefore, the high specificity of MOF-based and surface-modified materials provides a critical advantage in these applications, ensuring the reliability and accuracy of biosensing technologies in real-world scenarios.

The stability of 2D materials is another critical advantage, particularly in the context of biosensing applications. The chemical stability of MoS_2_, for instance, allows it to maintain its structural integrity and functional properties across a range of complex chemical environments. These include biological samples with varying pH levels (acidic or alkaline) and the presence of diverse biological enzymes and electrolytes [47]. Such stability ensures the reliable performance of MoS_2_-based biosensors in biological settings, where the material is often exposed to dynamic and challenging conditions. Similarly, the biocompatibility of ZnO is a key attribute that enables its use in biosensing applications. ZnO exhibits minimal interaction with living organisms, thus avoiding adverse immune responses or biotoxicity. This biocompatibility ensures that ZnO-based biosensors can operate stably over extended periods without inducing harm to biological systems, making them suitable for long-term monitoring applications [88]. The inherent stability of these materials not only minimizes potential detection errors arising from changes in material properties but also contributes to the extended lifespan of biosensors [89,90]. Such durability enhances the cost-effectiveness of biosensor technologies by reducing the need for frequent material replacement or recalibration.

Two-dimensional materials are also distinguished by their remarkable versatility. A prime example of this versatility is MOFs, which exhibit a wide range of physicochemical properties, including fluorescence, magnetism, and electrochemical activity. MOFs with inherent fluorescence properties offer a powerful tool for real-time tracking of biomolecule binding events. By monitoring changes in fluorescence intensity and wavelength, MOFs can provide an intuitive and dynamic visualization of biomolecular interactions. This capability allows for the continuous observation of the binding process, which is invaluable for applications that require precise and time-resolved detection of biomolecules [91]. Furthermore, the structural and compositional flexibility of MOFs allows for the customization of their properties, enabling the specific detection of a wide variety of biomolecules or drug molecules. By fine-tuning the metal ions, ligands, or pore structures, MOFs can be designed to selectively bind with target molecules, catering to a broad range of detection requirements. This adaptability makes MOFs highly suitable for diverse applications in biosensing, ranging from disease biomarker detection to drug monitoring and environmental analysis [92,93]. In addition to their inherent versatility, 2D materials can be integrated with other advanced technologies, such as microfluidic chip technology [94] and nanotechnology [95], to further extend their capabilities. The combination of 2D materials with microfluidics, for example, allows for the miniaturization and automation of biosensing platforms, enabling more efficient, cost-effective, and high-throughput detection. Similarly, the integration of nanotechnologies, such as nanoparticles and nanostructures, can enhance the sensitivity, specificity, and functionality of biosensors, enabling them to detect biomolecules at extremely low concentrations. These synergies open up exciting possibilities for the development of advanced biosensing platforms that are not only highly efficient and accurate but also more user-friendly and adaptable to a wide range of biological and drug detection scenarios.

In addition, recovery rates are critical for evaluating the efficacy of these sensors in complex biological matrices [96]. Efficient extraction and recovery of target analytes from biological samples, such as urine and serum, have been achieved through optimized functionalization of 2D material-based sensors [97] In one study, molybdenum disulfide was employed in a fluorescence detection platform to accurately quantify pathological microRNAs, achieving recovery rates exceeding 95% in complex sample matrices [98]. Such high recovery rates highlight the reliability and practical utility of these sensors in real-world applications [99]. Reproducibility is another crucial parameter, as it ensures the reliability and consistency of sensor performance across multiple tests [100]. The intrinsic structural stability of 2D materials enables reproducible signal responses [101]. For example, a photoelectrochemical sensor utilizing a CdS/MoS_2_ heterostructure demonstrated a coefficient of variation of less than 5% across repeated measurements, even under different experimental conditions or operators, showcasing excellent reproducibility [102,103]. The utility of 2D materials extends beyond conventional sensor designs to advanced detection technologies. Surface-enhanced Raman spectroscopy (SERS), for instance, has been significantly enhanced through the integration of nickel–cobalt hydroxide and silver nanoparticles, enabling ultra-sensitive detection of specific analytes [104]. The synergistic interaction between 2D materials and nanostructures in such systems illustrates their potential for analyzing complex biological and environmental samples with unprecedented precision.

Two-dimensional materials not only overcome existing challenges but also set new benchmarks in detection limits, recovery efficiency, and reproducibility. With continued advancements, their integration into emerging detection technologies will expand their applications from laboratory-based research to practical, on-site scenarios. This convergence of theoretical innovation and practical implementation marks a transformative step forward in the evolution of biosensor technology, laying a robust foundation for addressing the challenges of modern doping detection.

## 3. Application of 2D Materials in Doping Detection

In the field of doping detection, 2D materials also show great application potential with their unique physical and chemical properties. Doping encompasses a wide variety of substances, including anabolic steroids, peptide hormones, stimulants, masking agents, and other performance-enhancing compounds [105]. Each class of doping possesses unique physiological functions and structural characteristics, necessitating the development of sensors with both high sensitivity and selectivity to achieve accurate and reliable detection [106,107,108]. Moreover, the detection of doping substances presents substantial challenges, including the intricate composition of biological samples [109], the ultra-low concentrations of target analytes [110], and the presence of various interfering background substances [111]. Recent advancements in biosensing technologies based on 2D materials have led to notable progress in the detection of doping substances. These materials offer an ideal platform for the development of sensors capable of specifically identifying trace amounts of doping substances in biological samples. The high surface-to-volume ratio and the abundance of active sites on the surface of 2D materials, such as MoS_2_, graphene, and MOFs, enable them to capture and interact with target molecules efficiently, enhancing the sensitivity and specificity of doping detection.

The detection mechanisms for different types of doping substances often rely on the precise molecular interactions between the doping substances and the surface of the 2D material. For example, in the case of anabolic steroids or peptide hormones, sensors can be designed to selectively bind these molecules through specific recognition sites, such as antibodies or functionalized ligands, which are integrated into the material structure. This selectivity minimizes interference from other biomolecules and improves the accuracy of the detection process. Furthermore, the integration of additional detection modalities, such as fluorescence, electrochemical signals, or SERS, can amplify the detection signal, facilitating the identification of even trace amounts of doping agents.

The application advantages of 2D materials in doping detection are substantial. These materials not only provide high sensitivity and specificity but also exhibit excellent stability in complex biological environments, which is crucial for ensuring reliable and consistent performance over time. Moreover, the tunable properties of 2D materials allow for the customization of sensors to target specific doping agents, making them versatile tools for detecting a broad range of substances in various biological matrices. The flexibility in sensor design also facilitates the development of portable, cost-effective, and high-throughput detection platforms, which are essential for large-scale doping control in sports and clinical settings.

In summary, 2D materials present a promising avenue for the development of advanced doping detection technologies. Their unique physical and chemical properties, combined with recent advancements in biosensing techniques, position them as powerful tools for the accurate, selective, and efficient detection of doping substances, ultimately contributing to the integrity of sports and public health initiatives.

### 3.1. Detection of Anabolic Steroids

**Figure 5:**
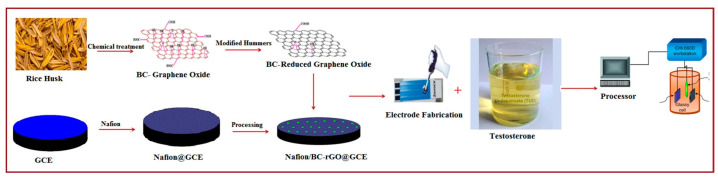
Electrochemical flow chart for the detection of testosterone based on BC-rGO. Reprinted with permission from reference [117].

Anabolic steroids, such as testosterone, nandrolone, and trenbolone, are commonly used performance-enhancing drugs that promote muscle growth by stimulating protein synthesis [112,113,114,115]. As a result, the detection of anabolic steroids, particularly synthetic testosterone, has become a critical focus in doping control. A novel composite material combining reduced graphene oxide (rGO) with MOFs has been developed for the detection of synthetic testosterone [116]. This material was synthesized through a tailored process that ensures effective integration of rGO with MOF structures. The rGO component provides excellent electrical conductivity, while the MOF contributes unique structural properties, enhancing the overall performance of the sensor. As shown in Table 1, the resulting rGO-MOF composite sensor exhibits remarkable sensitivity and selectivity, making it an effective tool for detecting anabolic testosterone in athletes’ biological samples. As shown in Figure 5, in a separate study, a biocarbon-derived porous reduced graphene oxide (BC-rGO) material was developed as a sensing platform for testosterone detection [117]. The BC-rGO material is a two-dimensional nanostructure characterized by a transparent layered structure and a rich folding dispersed throughout the entire substrate plane. Its synthesis from agricultural waste demonstrates an environmentally friendly scientific concept. When used to modify electrodes in a photoelectrochemical sensor, the BC-rGO material demonstrated exceptional sensitivity and selectivity for testosterone detection. The high sensitivity can be attributed to its unique porous structure, abundant oxygen-containing functional groups, and superior electrical conductivity, which facilitate efficient interaction with the target molecules and enhance the sensor’s response. This innovative sensor offers not only an effective and efficient method for detecting testosterone but also provides an environmentally sustainable alternative, which has significant potential in the field of biomedical detection.

### 3.2. Detection of Stimulants

**Figure 6:**
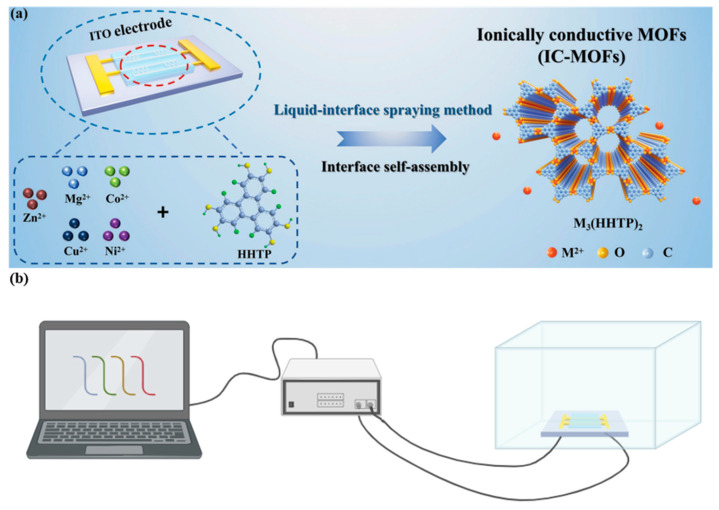
(**a**) Synthesis of Zn_3_(HHTP)_2_-MOFs and (**b**) flowchart of IC-MOF sensor detection. Reprinted with permission from reference [140].

The detection of stimulants, such as benzedrine and ephedrine, presents distinct challenges due to their unique structural characteristics and modes of action [118,119,140]. These substances require specialized detection strategies to achieve accurate and selective measurements. A nickel oxide–nitrogen-doped graphene oxide (NiO/NGO) nanocomposite was successfully synthesized through a hydrothermal method [119]. This material was used to modify a glassy carbon electrode (GCE), creating a novel sensor for the detection of ephedrine. The electrochemical catalytic activity of NiO, combined with the enhanced performance provided by nitrogen doping in the graphene oxide, resulted in a sensor with high sensitivity and excellent selectivity for ephedrine. The synergistic effect between NiO and nitrogen-doped graphene oxide significantly improved the electrochemical properties of the sensor, making it an effective platform for detecting ephedrine with high accuracy. Another advancement, shown in Figure 6, involves the development of ion-conducting metal–organic frameworks (IC-MOFs) [140], in which active metal anions are incorporated into layered MOFs with porous structures, allowing these anions to function as charge carriers. The resulting IC-MOFs demonstrated effective adsorption and binding capabilities, particularly for N-methylphenethylamine (MPEA). The sensor constructed from this material exhibited remarkable sensitivity for MPEA detection at room temperature, with an ultra-low theoretical detection limit of 20 ppt. The sensor also displayed rapid response times (approximately 5 s), excellent selectivity, and long-term stability.

These studies highlight the potential of advanced materials, such as IC-MOFs and NiO/NGO composites, in developing high-performance sensors for the detection of stimulants. The ability to achieve rapid, sensitive, and selective detection of substances like MPEA and ephedrine is critical for a variety of applications, including drug testing, forensic analysis, and public health monitoring. Moreover, the versatility and tunability of these materials make them suitable for further optimization, potentially enabling the detection of a broader range of stimulants and other performance-enhancing drugs.

### 3.3. Detection of Peptide Hormones

**Figure 7:**
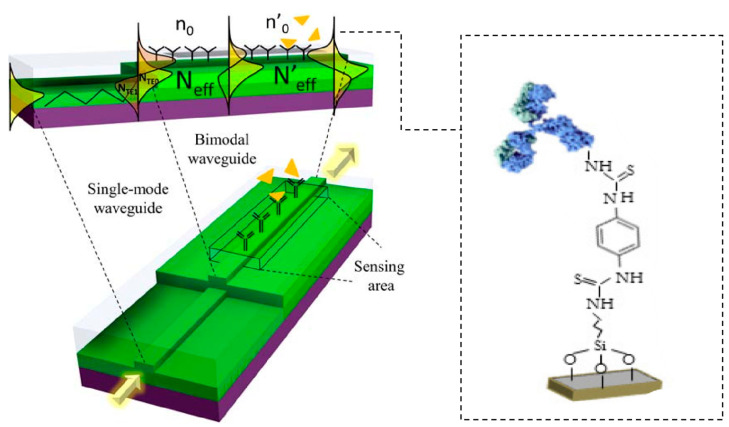
Schematic diagram of the sensing principle of a BiMW device. Reprinted with permission from reference [139].

Peptides hormones, as class S2 substances on the prohibited list, particularly erythropoietin (EPO) and human growth hormone (hGH), are among the most commonly abused substances in sports due to their performance-enhancing effects. EPO stimulates the bone marrow to produce more red blood cells, thereby increasing the oxygen-carrying capacity of the blood, which enhances endurance performance in athletes [141]. hGH, on the other hand, promotes muscle growth, improves strength, and accelerates recovery, making it particularly attractive in sports that require explosive power or high-intensity exertion [142]. Both EPO and hGH are banned substances due to their potential to undermine the fairness and integrity of competitive sports. Recent advancements in biosensing technologies have led to the development of innovative detection methods for these peptides. A notable example is the development of an electrochemical immunosensor based on nitrogen-doped reduced graphene oxide (N-rGO) and copper oxide (CuO) nanocomposites [143]. This sensor leverages the high electrical conductivity and large specific surface area of nitrogen-doped reduced graphene oxide, combined with the electrocatalytic properties of copper oxide, to enable highly specific detection of EPO. The sensor operates through antibody modification, which allows for the selective binding of EPO, showing a wide linear detection range and a low detection limit (as shown in Table 1) and facilitating its detection in complex biological samples. Figure 7 shows a biosensor based on a bimodal waveguide (BiMW) for the highly sensitive detection of hGH [139]. The sensor uses a waveguide structure consisting of a silicon nitride (Si_3_N_4_) waveguide core layer and a silicon dioxide (SiO_2_) cladding, fabricated with the aid of microelectronics, to provide high surface sensitivity and mode-specific optical behavior, along with the advantage of cost-effectiveness. The sensors utilize the evanescent field detection principle to achieve highly sensitive detection through changes in the refractive index triggered by the biometric process, leading to changes in the speed of light propagation in the waveguide. The bioreceptor layer is modified by anti-hGH antibodies and exhibits high specificity for target proteins, with no significant response to non-target hormones (e.g., hTSH). In the urine assay, the sensor exhibits low non-specific adsorption and good linear response for efficient detection of low concentrations of hGH, making it suitable for complex biological samples. In a dual-mode waveguide, artificially modified quasi-two-dimensional materials such as silicon dioxide may achieve higher sensitivity.

The studies listed in Table 1 highlight the great potential of 2D materials in the field of doping detection. Most of these materials are applied to graphene or graphene oxide electrodes for electrochemistry and to some MOFs. Although the application of 2D materials in this field is limited, the few reported properties show that they can achieve highly selective detection in complex biological samples. These properties can accurately identify trace target analytes in biological fluids, making them an ideal platform for the development of next-generation biosensors.

## 4. Biosensing Mechanisms for Doping Detection

### 4.1. Principles of Molecular Recognition

**Figure 8:**
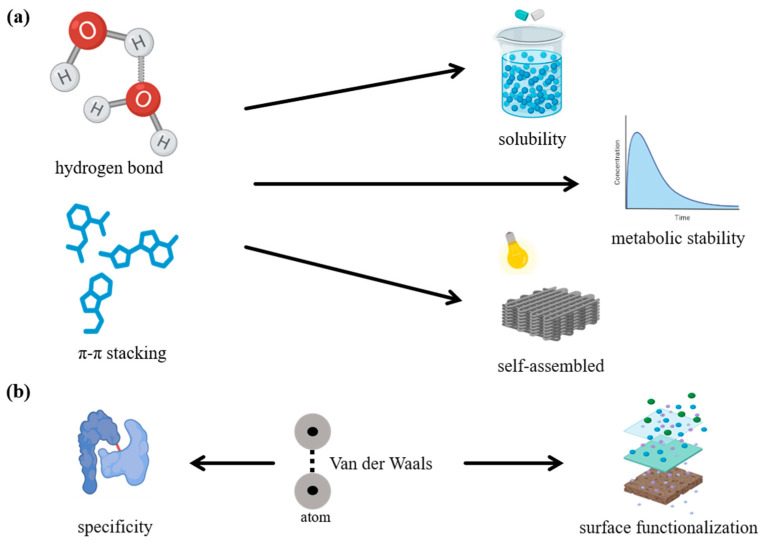
Functional schematic diagram of molecular recognition. (**a**) Hydrogen bonding and π-π stacking interactions play crucial roles in enhancing the precision and efficacy of molecular recognition, which is particularly advantageous for improving the solubility, bioavailability, and metabolic stability of drugs. (**b**) The synergy of electrostatic interactions and van der Waals forces further enhances the specificity of detection, while the ease of surface functionalization through van der Waals forces ensures that only target molecules bind, minimizing non-specific adsorption.

In the design of modern molecular recognition and drug delivery systems, the specific interactions between 2D materials and target molecules are fundamental to their functionality. As shown in Figure 8a, among these interactions, hydrogen bonding and π-π stacking interactions play particularly crucial roles in enhancing the precision and efficacy of molecular recognition [144]. Covalent organic frameworks (COFs), for example, possess surface-rich functional groups that are capable of forming hydrogen bonds with target molecules [145]. This interaction significantly improves the stability of molecule adsorption, especially for biomolecules containing hydroxyl or amino groups, thereby increasing the material’s affinity for these targets. These hydrogen bonding interactions exhibit a high affinity for molecules with aromatic structures, thereby enhancing both surface adsorption and the enrichment of target molecules [146,147,148], which is key to the effectiveness of molecular recognition systems. In drug delivery systems, π-π stacking interactions have emerged as a promising mechanism to enhance the stability and functionality of innovative materials, as well as to optimize the interactions between drug carriers and biomolecules [149]. These non-covalent interactions are particularly advantageous for improving the solubility, bioavailability, and metabolic stability of drugs. For instance, a study demonstrated that leveraging π-π stacking interactions between therapeutic molecules and carrier materials can significantly enhance the solubility and pharmacokinetic profiles of drugs [150]. Moreover, this approach enables precise modulation of drug release kinetics, facilitating controlled and sustained drug delivery, which is critical for achieving therapeutic efficacy and minimizing side effects [151]. In the context of 2D COFs, π-π stacking interactions play a pivotal role in improving both the structural and functional properties of these materials. The presence of π-conjugated systems within COFs enhances their electrical conductivity and biocompatibility. One study successfully synthesized COFs with highly ordered π-conjugated architectures, resulting in superior electron transport properties [152]. Additionally, the self-assembly characteristics and chemical stability conferred by π-π interactions enable COFs to perform reliably in challenging environments, such as those encountered in physiological and pathological conditions. These properties render COFs particularly promising for the development of smart drug release systems, where precise control over release rates and targeting is essential. Consequently, the integration of π-π stacking interactions within drug delivery frameworks provides a robust foundation for the development of next-generation materials with enhanced stability, functionality, and versatility. A recent study on π-stacking isomers provided valuable insights into the variations in π-π gaps and stacking orientations among different isomers, revealing the significant impact of these structural differences on the design of advanced drug delivery materials [153]. This phenomenon is particularly relevant for engineering multifunctional carriers, as it enables precise tuning of their chemical and physical properties by adjusting molecular stacking distances and angles. Such fine control facilitates enhanced drug loading capacities and greater adaptability of the carriers in diverse biological environments, paving the way for more effective and versatile therapeutic solutions.

Additionally, the synergistic effects of electrostatic interactions and van der Waals forces further enhance the specificity of detection (Figure 8b), ensuring that only the target molecules are bound, while minimizing non-specific adsorption [154]. Beyond their ability to precisely capture target molecules, these materials also maintain excellent biocompatibility, which is crucial for repeated use in biological systems without causing adverse effects. By integrating multiple 2D layers through van der Waals interactions, researchers have achieved novel physical properties, including improved mechanical strength and thermal stability. These multilayered structures offer an ideal framework for embedding and stabilizing drug molecules, while simultaneously enabling finely tuned, controllable release profiles [155]. Furthermore, some studies have focused on quantifying the interfacial adhesion forces of 2D materials and their van der Waals heterostructures under physiological conditions. This line of research aims to develop more practical and efficient drug delivery systems capable of targeted delivery and sustained release of therapeutic agents in complex in vivo environments [156]. Leveraging these enhanced interfacial adhesion characteristics, drug carriers can ensure precise targeting and minimize off-target effects, thereby improving therapeutic outcomes and reducing adverse reactions.

In terms of functionalization (Figure 8b), the surfaces of 2D materials are highly amenable to modification that can enhance their selective adsorption and sensing performance. A recent study highlighted that such surface functionalization not only enhances the material’s affinity for particular molecules but also facilitates the regulation of these interactions, allowing for more controlled and efficient detection processes [157]. By incorporating specific functional groups, it is possible to optimize the material’s performance for a broad range of applications, including the detection of biomolecules, environmental monitoring, and medical diagnostics. Two-dimensional materials hold immense potential for advancing the development of highly sensitive, selective, and versatile biosensors that can meet the diverse needs of modern scientific and medical applications [158].

Another innovative approach involves the use of light-driven self-assembly technologies, which incorporate light-responsive groups into 2D materials. This strategy enables dynamic modulation of drug carriers through light-induced processes, allowing on-demand activation of drug release and real-time adjustments to carrier morphology [159]. Such light-controlled mechanisms significantly enhance the flexibility and efficiency of drug transport, providing a robust platform for the development of intelligent drug delivery systems.

In conclusion, advancements in understanding and applying π-stacking interactions, van der Waals forces, and light-responsive self-assembly have opened new avenues for the development of next-generation drug delivery systems. These innovations hold immense potential for enhancing therapeutic precision, optimizing drug delivery efficiency, and tailoring treatments to individual patient needs, while also facilitating the development of doping testing.

### 4.2. Signal Transduction Mechanisms

**Figure 9:**
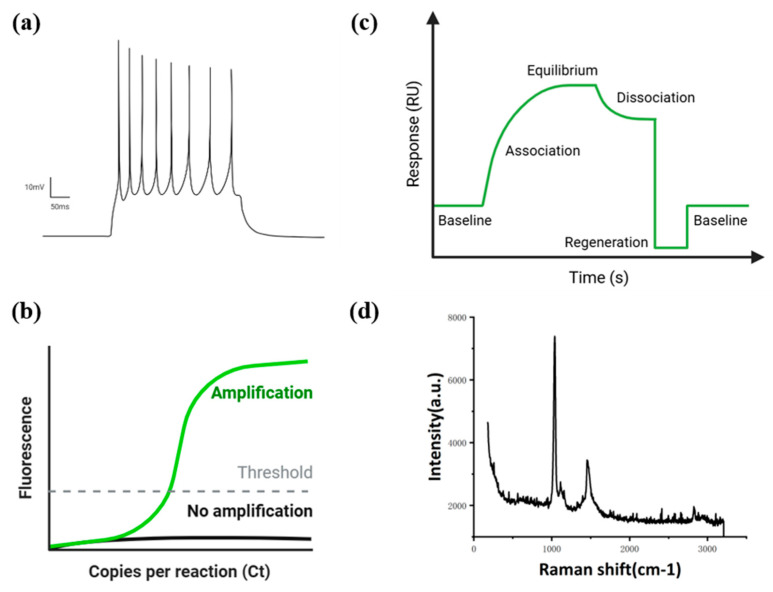
Schematic diagrams of signal transduction modes. (**a**) The figure shows an electrical signal, which is observed by changes in voltage or other parameters, such as resistance and capacitance. (**b**) The figure shows the intensity of fluorescence signals, which varies with the concentration of the analyte. (**c**) The figure is a schematic diagram of the change in the refractive index of a metal film surface used to detect the refractive index of a sample on the metal substrate. (**d**) The figure shows the detection of the concentration of a sample through the intensity of Raman signals.

In the field of biosensors employing 2D materials, particularly for doping detection, 2D materials demonstrate exceptional potential due to their unique physicochemical properties. Electrochemical and optical sensing technologies based on these materials offer substantial advantages in sensitivity, selectivity, and portability, presenting innovative solutions for detecting trace substances in complex biological and chemical environments. This review examines the specific performance characteristics and sensing mechanisms of these materials, highlighting findings from various research efforts in Figure 9.

From the perspective of electrical signal transduction, the interaction of doping molecules with 2D materials, such as graphene, induces alterations in the material’s charge distribution. This interaction results in measurable changes in electrical parameters, including resistance and capacitance [160]. Graphene, renowned for its ultra-high conductivity and large specific surface area, has been shown to exhibit extraordinary sensitivity to electrical perturbations caused by molecular adsorption [161]. By monitoring variations in conductivity, it is possible to quantitatively analyze the number of molecules adsorbed onto the surface, allowing for rapid and direct detection of doping concentrations through resistance fluctuations [162].

Beyond electrical detection, molybdenum disulfide (MoS_2_) has been extensively studied for its fluorescence quenching properties [163]. When MoS_2_ nanosheets interact with specific target molecules, the fluorescence intensity decreases proportionally to the concentration of the molecules, enabling highly sensitive quantification [164]. This fluorescence quenching mechanism offers an effective optical signal transduction pathway particularly suited for detecting doping in complex biological fluids and environmental matrices.

Surface plasmon resonance (SPR) sensing technology represents another advanced optical technique with significant applicability to doping detection [165,166]. By measuring changes in the refractive index at a metal film’s surface, SPR sensors enable real-time, label-free monitoring of molecular interactions. Studies have demonstrated that modifying metal surfaces with 2D materials can markedly enhance the sensitivity and selectivity of SPR-based sensors [167]. This enhancement facilitates the detection of small-molecule doping and provides novel methodologies for studying their interactions with biomacromolecules [168].

Raman spectroscopy also plays a pivotal role in characterizing and employing 2D materials for sensing applications. When 2D nanomaterials interact with target molecules, the resulting changes in Raman scattering signals allow both quantitative and qualitative analysis of the molecules [169]. Optimization of the interfaces of graphene and MoS_2_ has been shown to significantly enhance Raman signal intensities, improving detection sensitivity [97]. This capability positions Raman spectroscopy as a powerful tool for rapid and precise detection of illicit drugs and doping, offering extensive potential for future applications.

The diverse sensing mechanisms enabled by 2D materials, ranging from electrical conductivity changes to optical signal transduction, have substantially improved the efficiency and accuracy of doping detection. Advances in material synthesis and sensor design have further expanded their applicability, allowing for the development of versatile and high-performance sensing platforms. These innovations not only enhance the establishment of highly sensitive detection systems but also provide robust tools for trace detection in complex environments.

### 4.3. Factors Affecting Sensitivity and Selectivity

Various factors critically influence the sensitivity and selectivity of doping detection systems based on 2D materials, including their crystal structure, defect density, surface functionalization, and environmental parameters [170]. Extensive studies have been conducted to elucidate and optimize these aspects, significantly advancing the application potential of such materials in sensing technologies.

The crystal structure of 2D materials has been identified as a key determinant of their electronic and sensing performance. For example, high-crystalline-quality graphene exhibits superior electronic conductivity due to the formation of uninterrupted conductive pathways, which enhances both the intensity and stability of sensing signals [171]. This characteristic is essential for achieving rapid and reliable doping detection. In contrast, the deliberate introduction of controlled defects into 2D frameworks has been shown to create additional active sites, thereby improving the adsorption and molecular recognition capabilities of the materials [172]. Similar mechanisms have been reported in MOF studies [173], where moderate defect incorporation enhances functionality. However, excessive defects can compromise the mechanical integrity and conductivity of the material, particularly under high-stress conditions, underscoring the need for precise defect engineering [174].

Surface modification represents another critical factor in tailoring the selectivity of sensors [175]. By introducing specific functional groups, 2D materials can achieve precise recognition and effective binding of target molecules. For instance, functionalization of 2D materials with tailored moieties has been shown to significantly enhance selectivity [176]. Similarly, the modification of zinc oxide nanowires with organ silane molecules enabled the selective detection of acetone while suppressing interference from other gases [177]. These findings highlight the potential of surface engineering to fine-tune material interactions with specific analytes, thereby advancing sensor performance in complex detection environments.

Physicochemical conditions such as pH, temperature, and ionic strength play a pivotal role in modulating the performance of 2D material-based sensors. A study on material surface charge dynamics revealed that variations in pH directly influence molecular adsorption capacity, with optimized pH conditions significantly improving sensor sensitivity [178]. Similarly, adjustments in ionic strength have been shown to enhance the adsorption properties of 2D materials [179], thereby further optimizing detection performance. Regarding temperature effects [180], investigations into SPR sensors indicate that temperature fluctuations can substantially impact signal intensity and detection limits, emphasizing the necessity for rigorous temperature control during sensor operation.

Beyond material-specific factors, the optimization of detection devices and application environments has proven critical for real-world implementation. For instance, research on the on-site detection of fentanyl demonstrated that tailoring device conditions to specific environmental parameters significantly improved detection accuracy and response time [181]. Such advancements bridge the gap between laboratory research and practical applications, facilitating the development of portable, efficient detection systems for real-world scenarios.

In summary, the integration of optimized crystal structures, defect engineering, surface functionalization, and environmental adjustments has markedly enhanced the sensitivity and selectivity of doping detection systems based on 2D materials. Future research directions include the holistic integration of these factors to develop robust and efficient sensing platforms. Furthermore, translating these advancements into practical applications, such as drug testing, environmental monitoring, and health diagnostics, will have far-reaching implications for public safety and healthcare. With ongoing advancements in materials science and the evolving demands of detection technologies, this field is poised for transformative breakthroughs, enabling the development of next-generation sensing systems with unprecedented precision and reliability.

## 5. Challenges and Prospects

Although nanomaterials have shown great potential in pharmaceutical and electrochemical applications, their accuracy and stability in actual application scenarios still face considerable challenges. Especially in the application of two-dimensional materials, there is still controversy over whether the specific force by which they bind to the target substance is stable enough and whether they can effectively identify and detect the target substance. Therefore, for quantitative detection methods based on two-dimensional materials, further efforts are still needed to achieve breakthroughs in stability and sensitivity.

There are also many areas that can be optimized for materials of various forms. One of the foremost issues is the environmental stability of 2D materials. Susceptibility to oxidative degradation and hydrolysis under ambient conditions can compromise their structural integrity and sensing performance, thereby limiting their reliability and longevity in real-world applications [182]. Moreover, interfering substances present in complex biological matrices, such as proteins and metabolites [183], can compete with target doping molecules for active binding sites on the sensor surface. Such interference not only diminishes the selectivity of the sensors but also amplifies background noise, leading to distorted detection outcomes. Additionally, the high production costs associated with the synthesis of high-quality 2D materials, coupled with the financial burden of integrating and maintaining sensor systems, pose significant economic challenges that hinder large-scale deployment.

To address these limitations, several research directions hold promise. Enhancing the environmental stability of 2D materials through chemical modification or composite engineering can mitigate degradation [184]. For instance, introducing robust functional groups or incorporating metal ion complexes can fortify the materials against oxidative and hydrolytic effects [185]. Multimodal detection technologies represent another critical avenue for improving detection reliability [186]. By integrating electrical, optical, and magnetic signal modalities, multimodal platforms can achieve heightened resistance to interference while enhancing sensitivity to a broader range of analytes [187]. This integrated approach offers a robust framework for comprehensive data validation and analysis. Furthermore, reducing production and application costs is essential for scalability. Innovations in material synthesis, such as the adoption of cost-effective, sustainable processes, and leveraging economies of scale can significantly lower the economic barriers to widespread implementation.

Two-dimensional material-based detection methods exhibit distinct advantages, including reduced analysis time, simplified sample preparation, and decreased procedural complexity [188]. These characteristics make them particularly suitable for rapid detection and preliminary screening in high-throughput or time-critical settings [189]. The portability of such sensors further facilitates their application in field settings, such as in doping tests for athletes, where on-site detection is paramount [190]. However, conventional chromatography–mass spectrometry techniques remain the gold standard for qualitative and quantitative analyses, offering unparalleled accuracy, high resolution, and versatility in handling complex sample matrices [191]. These attributes are critical for detailed component analysis, particularly in scenarios involving matrix effects and interference from complex biological backgrounds [192,193]. Given the complementary strengths of nanotechnology-based sensors and traditional MS methods, their integration represents a promising research direction. Hybrid approaches that combine the rapid screening capabilities of 2D material-based sensors with the high-resolution analysis of MS can harness the advantages of both techniques. For example, recent studies have demonstrated that preliminary screening with 2D material-modified sensors, followed by detailed analysis with MS, significantly improves the overall sensitivity, specificity, and efficiency of stimulant detection [194,195]. Such integrative platforms provide a balanced solution, ensuring rapid initial screening while maintaining the precision required for confirmatory testing.

The integration of 2D materials with advanced technologies such as deep machine learning may further enhance the capabilities of biosensing platforms, particularly in the context of doping detection [196]. Machine learning algorithms have the potential to improve the efficiency and accuracy of detection by enabling real-time data analysis and decision-making. With ongoing technological progress and the expanding range of application scenarios, biosensors based on 2D materials are poised to move beyond laboratory settings, with potential applications in on-site detection [197], biomedical monitoring [198], and other critical areas of public health and sports safety.

In summary, there is still great potential for the development of doping detection technologies using two-dimensional materials at the nanoscale. Future researchers are required to fully understand the biosensing mechanism and combine it with precise optimization of material properties and innovative detection methods. At the same time, the inherent complexity and variability of the detection environment also need to be taken into account. This requires promoting interdisciplinary collaboration and building bridges between materials science, analytical chemistry, bioengineering, and computational modeling. Such collaboration can drive technological innovation, thereby developing next-generation detection platforms with better performance and a wider range of applications. By integrating these materials into advanced detection frameworks, the scientific community can contribute to maintaining the fairness of competitive sports, ensuring fair competition for athletes, and promoting public health and well-being. These emerging materials are not only a technological milestone but also demonstrate the transformative potential of nanotechnology in solving major social challenges.

## Figures and Tables

**Table 1:** Application of 2D materials for detecting different types of doping.

Sensor	Doping	Doping Category	LOD	Linear Range	RSD	Recovery Rate	Reference
2D AuNCs@521-MOF	Cocaine	S6	1.29 pM	0.001–1.0 ng·mL	0.02%	101.2–112.7%	[30]
NiO–GO/GCE	Pethidine (PTD)	S7	3.8 ng/mL	0–500 μg/mL	5.41%	90.00–99.66%	[76]
ZnO/GO/MOF	Nandrolone (NDL)	S1	0.09 µM	0.5 µM–138 µM	4.18%	94.00–99.00%	[113]
Pt-NPs@MOFs/Au-ZnO	Trenbolone (TB)	S1	3.61f g/mL	10 fg/mL–100 ng/mL	2.3–4.5%	96–107%	[114]
rGO/GCE	Testosterone	S1	0.1 nM	2.0–210.0 nM	1.97%	98.1–104.2%	[116]
Nafion–Gr/GCE	Caffeine *	---	0.12 µM	0.4–40 μM	5.20%	98.6–102.0%	[118]
NiO/NGO	Ephedrine (EPD)	S6	0.09 μM	10–2580 μM	3.591–4.21%	98.80–99.40%	[119]
Tb_4_O_7_/RGO	Salbutamol (SBM)	S3	21 nM	1 μM–710 μM	4.17%	96.50%	[120]
CTAB/ZnO	Carteolol Hydrochloride (CH)	P1	0.2 μg/mL	1 × 10^−3^–2 × 10^−1^ mg/mL	1.97–4.29%	96.5–110.5%	[121]
Ag-rGOPE	Triamcinolone Acetonide (TAA)	S9	0.005 μM	10–300 μM	4.37%	96.00%	[122]
Gr-CuO	Prednisolone	S9	0.008 µM	0.01–25 µM	3.40%	97.1–103.5%	[123]
rGO/GCE	Bumetanide (BMT)	S5	75 nM	0.255–50.0 μM	3.40%	99.3–101%	[124]
GO-CTAB-AuNP	Methamphetamine (MAMP)	S6	28.6 ng/mL	0.5–100 μM	4.70%	74.6–84.0%	[125]
magGO/PDA	Amphetamine-type stimulants (ASTs)	S6	0.272 μg/L	2–100 μg/L	0.9–6.0%	85.7–121.9%	[126]
GO/p-(AHNSA)	Atenolol (ATN)	P1	20 nM	0.1–300 μM	2.70%	97%	[127]
CeO_2_/rGO	Methamphetamine (MAMP)	S6	8.7 µM	25.0–166.6 µM	4.70%	——	[128]
Eu(H_4_BDPO)-MOF	Trenbolone (TB)	S1	2.36 fg/mL	10 fg/mL–100 ng/mL	3.20%	96.7–103%	[129]
Eu-MOF (Eu_2_[Ru(dcbpy)_3_]_3_)	Trenbolone (TB)	S1	4.83 fg/mL	5 fg/mL–100 ng/mL	2.70%	98.3–103.3%	[130]
C_60_/MWCNTs-Gr-IL/ITO	Nandrolone Decanoate (ND) Testosterone Decanoate (TS)	S1	——	2.12–11.38 Μm 1.03–7.75 μM	3.08% 3.33%	97–106% 96.3–103%	[131]
PtNPs/AuNWs/IL/CrGO	Metandienone	S1	0.4 nM	2 nM–9.6 μM	7.70%	95.70%	[132]
HLM/Au@RGO-CS/GCE	Testosterone	S1	4.2 mM	25–200 mM	3.13%	——	[133]
GNP/GCE	Dexamethasone (DEX)	S9	15 nM	0.1–50 μM	0.40%	——	[134]
CuO-Gr/CPE	Caffeine (CA)	---	0.01 μM	0.025–5.3 μM	3.70%	96.5–98%	[135]
ERGO	Caffeine (CAF)	---	0.055 μM	50–650 μM	5.00%	103.00%	[136]
PAN/Zein-rGO	IGF-1	S2	55.72 fg/mL	1 pg/mL–10 ng/mL	0.03%	95.81–101.54%	[137]
AuNPs@rGO-SnS	AgIGF-I	S2	0.12 pg/mL	0.25–750.0 pg/mL	3.12%	96.86–123.55%	[138]
BiMW/Si_3_N_4_/SiO_2_	hGH	S2	0.30 pg/mL	3–10 pg/mL	——	——	[139]

* Caffeine has not been on the Prohibited List since 2004, but its use is still monitored.

## Abbreviations

The following abbreviations are used in this manuscript:

LC-MS	Liquid chromatography–mass spectrometry
GC-MS	Gas chromatography–mass spectrometry
2D	Two-dimensional
MOFs	Metal–organic frameworks
WADA	World Anti-Doping Agency
OLEDs	Organic light-emitting diodes
Mo	Molybdenum
S	Sulfur
ZnO	Zinc oxide
SERS	Surface-enhanced Raman spectroscopy
rGo	Reduced graphene oxide
BC-rGO	Biocarbon-derived porous reduced graphene oxide
NiO/NGO	Nickel oxide–nitrogen-doped graphene oxide
GCE	Glassy carbon electrode
IC-MOFs	Ion-conducting metal–organic frameworks
MPEA	N-methylphenethylamine
EPO	Erythropoietin
Hgh	Human growth hormone
N-rGO	Nitrogen-doped reduced graphene oxide
CuO	Copper oxide
Si_3_N_4_	Silicon nitride
SiO_2_	Silicon dioxide
COFs	Covalent organic frameworks
SPR	Surface plasmon resonance
